# Causal influences of salience/cerebellar networks on dorsal attention network subserved age-related cognitive slowing

**DOI:** 10.1007/s11357-022-00686-1

**Published:** 2022-11-19

**Authors:** Clive H. Y. Wong, Jiao Liu, Jing Tao, Li-dian Chen, Huan-ling Yuan, Mabel N. K. Wong, Yan-wen Xu, Tatia M. C. Lee, Chetwyn C. H. Chan

**Affiliations:** 1 Department of Psychology, The Education University of Hong Kong, New Territories, Tai Po, Hong Kong China; 2 State Key Laboratory of Brain and Cognitive Sciences, The University of Hong Kong, Pokfulam Hong Kong, China; 3 Laboratory of Neuropsychology and Human Neuroscience, Department of Psychology, The University of Hong Kong, Pokfulam Hong Kong, China; 4 National-Local Joint Engineering Research Center of Rehabilitation Medicine Technology, Fujian University of Traditional Chinese Medicine, Fuzhou, Fujian China; 5 Fujian Key Laboratory of Rehabilitation Technology, Fuzhou, Fujian China; 6 Traditional Chinese Medicine Rehabilitation Research Center of State Administration of Traditional Chinese Medicine, Fujian University of Traditional Chinese Medicine, Fuzhou, Fujian China; 7 College of Rehabilitation Medicine, Fujian University of Traditional Chinese Medicine, Fuzhou, Fujian China; 8 Fujian Collaborative Innovation Center for Rehabilitation Technology, Fuzhou, Fujian China; 9 Department of Rehabilitation Sciences, The Hong Kong Polytechnic University, Hung Hom Hong Kong, China; 10 Department of Rehabilitation Medicine, Affiliated Hospital of Soochow University, Wuxi, Jiangsu, China

**Keywords:** Processing speed, Brain network, Effective connectivity, Automaticity, Effortful control

## Abstract

Age-related cognitive slowing is a prominent precursor of cognitive decline. Functional neuroimaging studies found that cognitive processing speed is associated with activation and coupling among frontal, parietal and cerebellar brain networks. However, how the reciprocal influences of inter- and intra-network coupling mediate age-related decline in processing speed remains insufficiently studied. This study examined how inter- and intra-brain network influences mediate age-related slowing. We were interested in the fronto-insular salience network (SN), frontoparietal dorsal attention network (DAN), cerebellar network (CN) and default mode network (DMN). Reaction time (RT) and functional MRI data from 84 participants (aged 18–75) were collected while they were performing the Arrow Task in visual or audial forms. At the subject level, effective connectivities (ECs) were estimated with regression dynamic causal modelling. At the group level, structural equation models (SEMs) were used to model latent speed based on age and the EC mediators. Age was associated with decreased speed and increased inter-network effective connectivity. The CN exerting influence on the DAN (CN → DAN EC) mediated, while the SN → DAN EC suppressed age-related slowing. The DMN and intra-network ECs did not seem to play significant roles in slowing due to ageing. Inter-network connectivity from the CN and SN to the DAN contributes to age-related slowing. The seemingly antagonizing influences of the CN and SN indicate that increased task-related automaticity and decreased effortful control on top-down attention would promote greater speed in older individuals.

**Supplementary Information:**

The online version contains supplementary material available at 10.1007/s11357-022-00686-1.

## Introduction 

Cognitive ability begins to decline from early adulthood [1]. The yardstick for such decline is the slowing of cognitive processing speed (PS), which refers to how fast information is detected, encoded and processed. The time required to complete the processes described has been reported to increase with individuals’ age [2, 3]. Previous studies have focused on the changes in the neural substrates and their networks due to neurodegeneration such as grey matter volumetric reduction and white matter pathologies [4]. However, how the interactions among these substrates and networks would influence the slowing manifested in behavioural performance has not been sufficiently studied. The current study is aimed at examining the neural dynamics within and between the PS-related brain networks to explain the increasing slowing due to normal aging using functional brain imaging.

PS is often measured with tests ranging from simple reaction time tasks to complex psychometric tests. Although these tasks/tests demand different cognitive abilities, earlier statistical studies revealed a latent speed factor which is independent of the task-taking processes [5], suggesting a substantial task-generic component. Similarly, functional magnetic resonance imaging (fMRI) studies have demonstrated that diverse cognitive demands recruited several core neural systems [6, 7], including the fronto-insular salience network (SN) [8], frontoparietal dorsal attention network (DAN) [9] and cerebellar network (CN) networks [10], and deactivated the default mode network (DMN) [11]. Cole et al. [12] found that the network architecture was almost invariant across different brain states. Notably, they concluded that “small but consistent changes common across tasks suggests the existence of a task-general network architecture distinguishing task states from rest”. Hence, primitive cognitive tasks could tap into the task-general networks that play an essential role in cognitive slowing.

Age-related changes in functional connectivity (FC) were observed in both intra- and inter-network connections among SN, DAN, DMN and CN [13, 14]. Archer et al. [15] reported reduced intra- but increased inter-network connectivity of SN among older adults. To our knowledge, only one study has documented the mediation effect of FC on age-related slowing of visual PS [16]. They found that reduced within-network FC of the SN mediated the age-related decline of visual PS. Since SN regulates the activities of other networks, they postulated that disruption of this function leads to cognitive slowing. However, intra-SN connectivity was not sufficient to justify their claim; the influence of SN on other networks also played a role. Furthermore, inter-network connectivities of CN were associated with slower PS among older [17] and younger adults [18]. In particular, after isolating cognitive PS from perceptual-motor speed, Wong et al. [18] found that task-related SN → DAN and CN → DAN effective connectivities (ECs), which measure the functional influence of one region on another, were related to PS. We proposed that the interactions between cognitive control (subserved by SN) [19] and automated cognitive processes (subserved by CN) [20] influence the speed of the task-taking process [21]. Since effortful control and automaticity supported processing speed in younger adults, the age-related deterioration and compensation mechanisms should be closely related to those systems.

The current study aimed to use fMRI to evaluate the effect of inter- and intra-network ECs on age-related slowing with structural equation modelling (SEM). In particular, we were interested in testing the connective networks across the frontal, parietal and cerebellar regions. Different methods have been deployed to study brain networks. Besides fMRI, electroencephalogram (EEG) and functional near-infrared spectroscopy (fNIRS) are the candidates. Recent studies demonstrated the feasibility of reproducing brain networks with EEG [22] and fNIRS [23]. Researchers also developed enhanced algorithms such as the channel-based [24] and source-based [25] methods to establish inter- and intra-network measures. We preferred to use fMRI over EEG and fNIRS because of two reasons. Firstly, fMRI has a superior resolution power than the other two methods, especially for reaching deep neural structures such as the cerebellum. Secondly, the handful of recent publications on the EEG and fNIRS limit the design and trial of the analytic algorithm. Other studies also commented that the evidence of the validity of EEG and fNIRS on measures of networks, including the insular and cerebellum, required further research [26–28]. The current study, therefore, employed fMRI to quantify brain activities. To rectify perceptual and motor confounds, we administered the Arrow Task, which involved two perceptual modalities, two levels of cognitive demand and two modality-matched simple reaction times (RTs) [18]. Since ECs across the SN and CN were associated with PS among younger adults [18], inter-network ECs among the SN, DAN, CN and DMN were examined. We hypothesized that the SN → DAN, CN → DAN and intra-SN ECs would mediate age-related cognitive slowing. Since previous studies covered the role of intra-network connectivities in cognitive aging [15–18], we also explored the contribution of intra-CN, -DAN and -DMN ECs.

## Method

### Participants

Healthy young (age: 18–28, *N* = 41), middle-aged (age: 45–55, *N* = 31) and older (age: 65–75, *N* = 41) adults participated in this study. After excluding participants with extensive head movement or premature and erroneous responses, or unable to complete the tasks inside the scanner, the final sample had 83 participants (young, 35, *M* = 20; middle-aged, 25, *M* = 11; older, 23, *M* = 10). Normal or corrected-to-normal vision and audition were screened with the E Standard Logarithm Eyesight Table and pure-tone detection test at 30–1000 Hz octave frequencies. All participants completed the Edinburgh Handedness Questionnaire [29], Montreal Cognitive Assessment, Beijing Version (MoCA) [30] and Hamilton Rating Scale for Depression [31]. They were right-handed and free from cognitive impairments (MoCA < 26), depressive mood (HAMD ≥ 7), neurological diseases, substance abuse, smoking and MRI contraindications. Informed consent was obtained before the experiment. Ethical approval was obtained from the Ethics Committee of the university where the study was conducted.

### Arrow Task

The Arrow Task was used in a prior fMRI study [18]. There were six conditions: three levels of cognitive demands (compatible, incompatible and control) and two perceptual modalities (audial and visual). In the two compatible (COM) conditions, the participants would press the “UP” button when viewing an up arrow (visual condition) or hearing a high-pitched sound (audial condition) and the “DOWN” button for a down arrow or a low-pitched sound. In the two incompatible (INC) conditions, the task rules for pressing the “UP” or “DOWN” buttons were reversed. In the two control (CON) conditions, the participant would press any button without considering the task rules. This three-by-two task design was meant to enable the possible partitions of the sensory and motor speeds in the data analyses [18]. The audial and visual conditions were delivered in two separate sessions; each session had 15 blocks (five blocks for each of the COM, INC and CON conditions), and ten trials made up each block. The blocks were randomized and counterbalanced. The task rules were presented at the beginning of each block. In each trial, the visual or audial stimulus was presented for 800 ms, followed by a fixation cross of 1000 ms in duration. The duration of each session was 350 s, and the entire experiment took 700 s. MRI scanning parameters and preprocessing steps are detailed in the Supplementary Method.

### Network and region of interest definitions

**Fig. 1:**
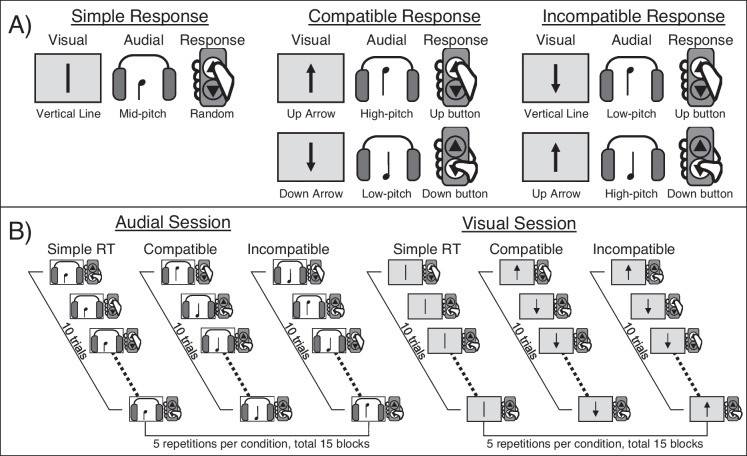
The Arrow Task. **A** Stimulus–response mapping for the three task conditions for each of the two modalities. **B** Experimental design. Trials were pseudorandomized within a block. Blocks were randomized at condition level (e.g. ABCABC…, BCABCA…). Randomization of blocks was counterbalanced at the group level

Extractions of the regions of interest (ROIs) for analyses were based on the results of three previous PS studies from the open repository NeuroVault [32]. In brief, a common activation map was calculated, a watershed method was applied to define the clusters, and the peak 150 voxels were extracted as the ROI (see Supplementary Method and Fig. 1B).

### Effective connectivity estimation—subject level

**Fig. 2:**
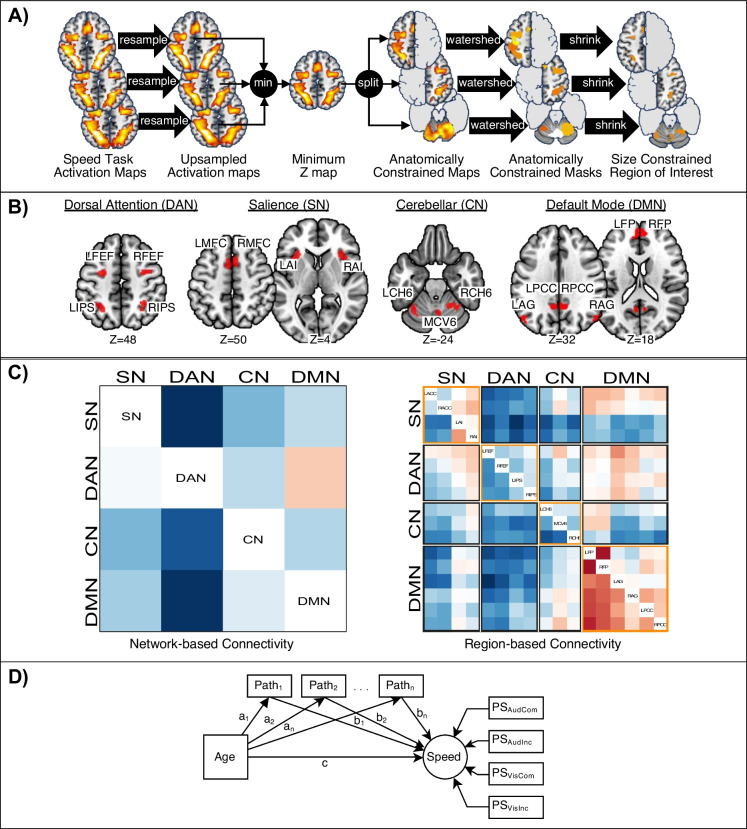
Procedures for establishing effective connectivity measures. **A** Extraction of regions of interest (ROIs) from previously published activation maps. Three activation maps of processing speed (PS) tasks obtained from a public repository were upsampled to isotropic 2-mm voxels, minimum filtered and split into left and right cortices and cerebellum. Clusters were obtained with the watershed method and shrunk to about 150 voxels. **B** Network assignment of the identified ROIs. Attention network, bilateral frontal eye-field (LFEF/RFEF) and intra-parietal sulcus (LIPS/RIPS); salience network, bilateral anterior cingulate cortex (LACC/RACC) and inferior frontal gyrus (LIFG/RIFG); cerebellar network, bilateral hemisphere and vermal lobule VI (RCH6/RCH6/MCV6); default mode network, bilateral frontal pole, angular gyrus and posterior cingulate cortices (LFP/RFP/LAG/RAG/LPCC/RPCC). **C** Illustration of network-level connectivity and region-level connectivity matrices established at the subject level. Network-level connectivity was estimated with the time series of the 4 networks, while region-level connectivity was estimated with the time series from 17 ROIs. For region-level connectivity, the 4 submatrices surrounded by orange boxes were within-network connections, while the other 12 were between-network ones. For example, there were 6 connections in the within-CN submatrix and 16 connections in the SN → DAN between-network submatrix. **D** Mediation models. Network-level connectivities (1 model) and region-level between-network connectivities (12 models) were submitted to parallel connectivity mediator models, and region-level within-network connectivities (4 models) were submitted to latent connectivity mediator models. SN, salience network; DAN, dorsal attention network; CN, cerebellar network; DMN, default mode network; LACC/RACC, left/right anterior cingulate cortex; LAI/RAI, left/right anterior insula; LFEF/RFEF, left/right frontal eye-field; LIPS/RIPS, left/right intra-parietal sulcus; LCH6/RCH6, left/right cerebellar hemisphere lobule VI; MCV6, medial cerebellar vermis VI; LFP/RFP, left/right frontal pole; LAG/RAG, left/right angular gyrus; LPCC/RPCC, left/right posterior cingulate cortex

We modelled the inter-network ECs among the derived SN, CN, DAN and DMN systems with regression DCM [33]. For each participant, the ECs were estimated as endogenous connectivity matrices (i.e. A-matrix). Analyses of the BOLD signals obtained from the audial and visual conditions were concatenated. The first eigenvalue from the network masks (AN, SN, CN and DMN) became the BOLD time series. The DCM model had four regressors, fully and bidirectionally connected and driven by the task conditions. The inter-network EC showed 12 causal pathways. The estimation of the intra-network EC was based on 17 regions (4 SN, 3 CN, 4 DAN and 6 DMN substrates). All the regions were included in one DCM model to avoid possible influences imposed by other networks [34]. The regional EC involved 272 causal pathways among the 17 regions. Among them, only four intra-network EC submatrices were extracted (Fig. 2C).

### Structural equation model—group level

For all the regression and SEM models, the independent and dependent variables are age and speed, respectively, with the former as a continuous observed variable and the latter as a latent variable with four PS indicators calculated from the RTs. There were four conditions: Audial COM (PS_AudCom_), Audial INC (PS_AudInc_), Visual COM (PS_VisCom_) and Visual INC (PS_VisInc_). The speed indicators were calculated as the negative standard score of the RT differences between COM and CON and between INC and CON. Higher values reflect a faster speed.

We tested age-speed (c-path) and age-EC (a-paths) associations as the prerequisite of the mediation model. Separate regression models were estimated for each of the inter-network and intra-network EC pathways. Significant pathways were then entered into subsequent SEM models as parallel mediators (Fig. 2D). A model was built for each of the inter-network and 4 intra-network EC sets. Age was modelled as a continuous independent variable, and four PS measures were loaded onto the latent speed variable. Parameter estimates and 95% confidence intervals of the modelled parameters were calculated with 5000 non-parametric bootstraps. We conducted power analysis with the intended models (see supplementary material for details). The current sample size (*N* = 83) showed adequate fit for the between-network, within-SN, within-DAN and within-DMN models and an acceptable fit for the within-CN network. All regressions and SEM analyses and power analyses were performed with the lavaan package version 0.6–9 and semPower package version 1.2.0 in R version 3.6.1.

## Results

### Regression: age-speed and age-EC associations

The latent age-speed regression (Supplementary Fig. 1) indicates a significant age-related decline in the PS (*β* =  − 0.535, 95%CI: [− 0.700, − 0.362]). The inter-network age-EC regressions show that only the influence of the SN, CN and DMN on the DAN system was significantly positively associated with age (SN → DAN: *β* = 0.403, 95%CI: [0.209, 0.567]; CN → DAN: *β* = 0.346, 95%CI: [0.171 0.507]; DMN → DAN: *β* = 0.398, 95%CI: [0.236, 0.538], Supplementary Fig. 2 and Supplementary Table 1). A similar pattern was observed among the inter-network regional ECs (Supplementary Fig. 3). The results indicate that the between-network ECs increased with age. Intra-network ECs revealed an interesting pattern (Supplementary Fig. 3). About half of those EC pathways demonstrated age-related changes; those significant ones within the SN, DAN and CN systems increased with age, while those within the DMN system decreased with age.

### Inter-network SEM model

**Fig. 3:**
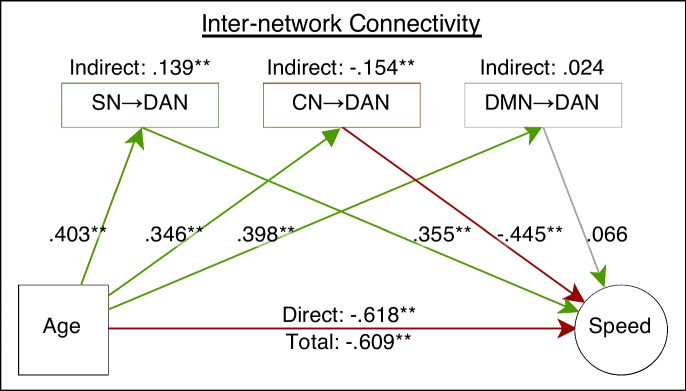
Path diagram of the mediation model with network-level connectivities as parallel mediators. Indicators of the latent speed variable, variance and covariance paths were omitted. Variables in rectangles and ovals were measured and latent variables, respectively. Connectivity mediators in green rectangles: positive mediator (SN → DAN). Connectivity mediators in red rectangles (CN → DAN): negative mediator. Connectivity mediators in grey rectangles (DMN → DAN): insignificant mediator. Green arrows: significant positive estimates. Red arrows: significant negative estimates. Grey arrow: insignificant estimates. Double asterisks: 95% CI did not contain zero. SN, salience network; DAN, dorsal attention network; CN, cerebellar network; DMN, default mode network

Among the 12 pathways, the influences whose destination is DAN (SN → DAN, CN → DAN and DMN → DAN) were significantly associated with age and were entered in the SEM model (Fig. 3, Table 1). The inter-network SEM model indicated a significant suppression effect for SN → DAN and a mediation effect for CN → DAN, while the DMN → DAN effect on speed was not significant. Age was negatively associated with speed (c’-path). The EC of these three pathways were all positively associated with age (a-paths: SN → DAN, CN → DAN, DMN → DAN). However, the established EC-speed association was significantly positive for the SN → DAN pathway, significantly negative for the CN → DAN pathway and non-significant for the DMN → DAN pathway (b-paths; SN → DAN, CN → DAN, DMN → DAN). The indirect effects between age and speed were SN → DAN pathway as a suppressor, CN → DAN pathway as a mediator and DMN → DAN pathway was not significant. The total effect of age on speed was also significant. The estimated post hoc power of the SEM analysis was 0.273. Computation of the power was based on one age variable, three effective connectivity mediators, four speed indicators, one covariance parameter among the mediators and four covariance parameters among the speed indicators.

**Table 1:** Inter-network mediation model

Parameter	Formula	β	95%CI
Latent speed	PS = ~ PS_AudCom_	0.654	**[0.371, 0.900]**
	PS = ~ PS_AudInc_	0.566	**[0.309, 0.817]**
	PS = ~ PS_VisCom_	0.599	**[0.380, 0.804]**
	PS = ~ PS_VisInc_	0.782	**[0.537, 1.088]**
a-paths	SN → AN ~ Age	0.403	**[0.208, 0.567]**
	CN → AN ~ Age	0.346	**[0.171, 0.507]**
	DN → AN ~ Age	0.398	**[0.236, 0.538]**
b-paths	PS ~ SN → AN	0.355	**[0.025, 0.626]**
	PS ~ CN → AN	− 0.445	**[− 0.759, − 0.090]**
	PS ~ DN → AN	0.066	[− 0.216, 0.321]
c-path	C	− 0.618	**[− 0.896, − 0.334]**
Indirect effects	SN → AN	0.139	**[0.011, 0.269]**
	CN → AN	− 0.154	**[− 0.300, − 0.025]**
	DN → AN	0.024	[− 0.096, 0.130]
Total effect	Total	− 0.609	**[− 0.833, − 0.356]**

Estimates of variance and covariance parameters were listed in Supplementary Table 3.

### Intra-network SEM models

No significant mediation effects were found for all the intra-SN, -CN, -DAN and -DMN models. (Supplementary Table 4–7).

## Discussion

The current study attempted to explain age-related slowing in terms of effective connectivity, or causal influence, among neural substrates. To our knowledge, the current study is the first to look into how inter- and intra-network influences contribute to the age-related slowing of processing speed. First, the results confirmed that only inter-network influences of SN → DAN, CN → DAN and SN → DMN increased with age. Therefore, it would be inter-network but not intra-network connectivity mediating age-related slowing. The CN → DAN was found to mediate slowing, which supports our hypotheses. The positive CN → DAN results indicate that cognitive slowing would be driven by increasing in the influence from the cerebellum to the top-down attentional system. Contrary to our hypothesis, however, the SN → DAN appears to mitigate cognitive slowing. The negative SN → DAN results suggest that an increase in influence from the cognitive control system to the top-down attention system would preserve speed during ageing. Our findings offer evidence of the interactions between the influences exerted by the salience network and the cerebellum in cognitive slowing. Interventions targeted at adjusting the balance between the two influences should have the potential to preserve the processing speed in older adults.

### Dorsal attention network

The influences from the SN and CN to the DAN significantly predicted participants’ processing speed. The “hub-like” phenomenon concurs with a recent study [35] that the DAN plays a role in the flow of information. In addition, our multi-modality task design supports the DAN processes- and modality-independent representations [36]. The extensive inter-network connectivity revealed, i.e. with SN and CN, further supports the reports of previous studies that processing speed was associated with activation [37] and coactivation [38] of the DAN.

### Cerebellar network driving age-related slowing

The most significant finding was the influence of the cerebellum on the DAN (CN → DAN), which contributed to age-related slowing. Associations between cerebello-cortical connectivity and processing speed have been found in younger [18, 39] and older [17] adults. Although it seems contradictory to frontal-dominated explanations such as the frontal aging hypothesis [40], posterior-anterior shift in aging [41] and scaffolding theory of aging and cognition [42], it corroborates a recent volumetric study [36]. In particular, volumetric covariations among the cerebellar and prefrontal substrates were associated with age-related slowing. Most importantly, the aforementioned [43] cerebellum component was topographically similar to the clusters identified in the current study. In addition to the speed-volume association, the current study’s findings revealed that the CN → DAN influence was excitatory across ages (Supplementary Table 9). In other words, the input from the cerebellum to the DAN drove activations of the attention system. Since the influence increased with age and was inversely related to speed, we purport that, among older adults, more cerebellar inputs play an important role in boosting the activities of the attention system in completing the cognitive process in time.

The significant neural substrates identified in this study are in the bilateral hemisphere and the vermis of lobule VI. Previous studies reported that the vermis VI is part of the cognitive cerebellum [44] supporting task-related timing and perception and automaticity [18]. We used a processing speed task that involved stimulus–response and task processes that are consistent with those subserved by lobule VI. The cerebellar internal forward model simulates prefrontal information processing with higher speed and accuracy but is less flexible compared to the cortex automatic process in coupling the response with the stimulus [45]. The cerebellum deteriorates across age, which underpins cognitive decline [46]. The increases in the influence from the CN revealed in this study imply that the slowed and inefficient internal model contributes to the slowing.

### Salience network antagonizing age-related slowing

Contrary to the CN → DAN, the age-related increases in the SN → DAN influence were associated with faster speed, while within-SN ECs were not significant. The SN consists of the anterior cingulate cortex and bilateral anterior insula [8]; it is responsible for the detection of bottom-up salient events and up- and downregulating other neural systems in response to the task goal [47, 48]. Contrary to our findings, Ruiz-Rizzo et al.’s study showed that the within-SN (reduced connectivity from left AI to the rest of the SN at rest) connectivities mediated age-related slowing [16]. The inconsistency may due to the methodological difference between task-based EC and resting-state FC. More importantly, we found that the involvement of SN in age-related slowing was via its influence on the DAN. These findings are consistent with a recent resting-state study that showed that older adults’ particularly slow processing speed was predicted by the inter- rather than intra-network connectivities [17]. Interestingly, the SN → DAN influence did not mediate but indeed mitigated the age-related slowing of speed. We postulate that the role of SN would be to maintain updating while upregulating the DAN’s activities to engage in the task, and the function of lower resting intra-SN FC in mediating [16] and higher task SN → DAN EC in suppressing cognitive slowing are two sides of the same coin. Further studies are warranted to validate this postulation on mitigating age-related slowing.

### Default mode network

The DMN → DAN hypothesis was not supported by the results. Previous studies reported that DMN connectivity was associated with an age-related decline in performance on executive control tasks such as those involving working memory [49] and inhibition [50]. The age-related DMN-DAN interaction was found only during an inhibition task, but not at rest [50]. Also, a previous study found that the volume of DMN was not associated with processing speed in the context of cognitive slowing [43]. In other words, the relationship between DMN and DAN is likely to be specific to higher cognitive demand.

### Limitations

Although EC can inform inter-network influences, the results of the present study are based on neural correlates and participants’ task performances. Future studies should use causal methods such as brain stimulation or performance training to gather more evidence on the inter-network phenomenon. This study employed simple stimulus–response mapping and cross-modality task conditions that yielded a latent speed variable. The task design was meant to minimize the drawback of task-specificity activations found in previous studies. However, the results generated from this study’s task conditions cannot be generalized to complex cognitive task designs. For instance, complex tasks may involve activations in the posterior rather than anterior parts of the cerebellum and hence modify the inter-network connectivity. The robustness of the functional couplings revealed should be further tested. The relatively high dropout rate, particularly among the older adult participants, would have reduced the generalizability of the results, and readers are reminded to interpret them with caution. In addition, the dropouts reduced the power of the analyses. Future study is to consider replicating the study with larger sample size. Lastly, the current research employed fMRI as the investigative method. This high-cost method would limit the potential applications of the connective network results in clinical situations. Future studies will explore the feasibility and develop enhanced data collection and analysis methods to replace MRI with EEG or fNIRS. The latter two ways are lower cost and more tolerable among the older participants.

## Conclusion

The current study examined how neural network connectivities mediate age-related decline in processing speed. The inter- but not intra-network connectivities showed that influences from the cerebellum and the salience network on the attention network regulated cognitive slowing. The salience network’s influence suppresses, while the cerebellar network’s influence mediates the age-related slowing. Age-related cerebellar influence imposes negative impacts on the top-down attention system. Cognitive training referencing the internal forward model may prevent older adults from deteriorating with respect to slowing. Practices that focus on performance accuracy and task-rule flexibility may be beneficial. Another important criterion in the practices is to achieve a set level of automaticity before moving up to a higher level. Moreover, the age-related salience network imposes a positive impact on mitigating slowing. This part of our findings also implicates training content. For instance, sensitivity and preciseness in detecting and encoding external information would improve age-related slowing. Clinical interventions can build on our previous research reporting the positive effects of audio-visual integration [51, 52] and how Chinese character encoding and manipulation [53] enhanced older adults’ attention and processing speed. Future studies are needed to gather evidence on the effectiveness of these interventions in enhancing processing speed in the older population.

## Supplementary Information

Below is the link to the electronic supplementary material.

Supplementary file1 (DOCX 1225 KB)
