# Supplementary material for: Causal influences of salience/cerebellar networks on dorsal attention network subserved age-related cognitive slowing
Source: GeroScience. 2022 Nov 19;45(2):889–99. doi: 10.1007/s11357-022-00686-1 (PMC9886783; doi:10.1007/s11357-022-00686-1)
Supplement: Supplementary file 1 — Supplementary file1 (DOCX 1225 KB) [file 11357_2022_686_MOESM1_ESM.docx]

**Causal Influences of Salience/Cerebellar Networks on Dorsal Attention Network Subserved Age-related Cognitive Slowing**

# Supplementary Method

## Representativeness of the Included Participants

Given that the dropout rate is 26.5% (40/113), there is a risk that the final sample was biased toward certain demographical factor. To this end, we examined if the demographics of the participants were associated with their inclusion. In particular, the effect of age and sex on the inclusion were tested with logistic regression models with adjustments on group. The effect of age (𝛘²(1,N=103)=0.058, p=0.810) and sex (𝛘²(1,N=103)=0.050, p=0.810) on inclusion was not significant, indicated that there were no significant bias of demographics on whether or not the participant is included in the final sample.

## MRI Scanning Parameters and Data Preprocessing

Anatomical and function images were acquired with GE Signa HDxt 3T scanner with 8-channel phased-array head coil. Anatomical images were acquired with MP-RAGE sequence (FOV=240 x 240mm, slice thickness=1mm without gap, 160 axial slices, acquisition matrix=256 x 256, TR/TE=5556/1764, inversion time=450, flip angle=15°) and functional images with EPI sequences (FOV=240 x 240mm, thickness=4mm without gap, 40 axial slices, acquisition matrix= 64 x 64, TR/TE=2000/30ms, flip angle=90°) from each subject.

Functional MRI sessions were ICA-cleaned with FSL/MELODIC (version 5.0.9 Jenkinson et al., 2012). Prior to independent component analysis, the first 5 volumes were removed, a 1/90Hz high-pass filter and a 5-mm FMHW Gaussian spatial filter were applied, images were aligned with the middle volume. Artifactual components were removed through visual inspection [1]. Field inhomogeneity correction, rigid co-registration and diffeomorphic normalisation were conducted with Advanced Normalization Tools [2].

## Region of Interests

Extraction of the region of interests (ROIs) for analyses was based on the results of three previous PS studies from the open repository NeuroVault [3]. The tasks were Letter Comparison, Pattern Comparison and Digit-Symbol Task. The minimum Z value was calculated after resampling the 3 mm to 2 mm isotropic voxels. Spatial-constrained masks were derived to avoid ROIs spanning through hemispheres and cerebellum. Minimum Z maps were then split into the left cortex, right cortex, and cerebellum. A watershed method was applied on the maps and 17 clusters were obtained. Cluster maps were shrunk to a uniform size of 150 voxels to obtain the ROIs, which were combined into four network masks. The SN was comprised of the bilateral anterior cingulate cortex (LACC, RACC) and anterior insular (LAI, RAI), the DAN was the bilateral frontal eye-field (LFEF, RFEF) and intraparietal sulcus (LIPS, RIPS), the CN was the bilateral lobule VI (LCH6, RCH6) and vermis VI (MCV6) and the DMN was the bilateral frontal pole (LFP, RFP), angular gyrus (LAG, RAG) and posterior cingulate cortex (LPCC, RPCC).

## Regression DCM

Traditionally, the B-matrix of the DCM was utilised to infer context-dependent modulation of effective connectivity. However, as the inter-network influences supporting processing speed should be task-invariant, the endogenous connectivity strength of the model was employed [4].

## Structural Equation Models

Covariances among the Speed and covariances among the mediators were modelled when the corresponding modification index was above 3.84, which was equivalent of the Chi-square p<0.05 of one degree-of-freedom [5]. The direct effect of Age on latent Speed variable ($c'$), the effect of Age on mediators ($a_{1}\cdots a_{n}$) and the effect of mediators on latent PS ($b_{1}\cdots b_{n}$) were estimated. Indirect effects ($m_{i}$) were calculated as $a_{i}\times b_{i}$ for each mediator and the total effect was calculated as the sum of $c'$ and all $m_{i}$.

## Sample Size Estimation with Power Analysis

We conducted several a-priori power analyses with the full model that specified one independent variable of age, a set of effective connectivity mediators, four speed indicators and no further specification of covariances among mediators and among indicators. We set the model to achieve an adequate fit (RMSEA: 0.05-0.08) or acceptable fit (RMSEA: 0.08-0.1, Hu & Bentner, 1999) at an alpha=0.05 and power=80%. There are 12 mediators for the between-network, within-DAN and within-SN network models. Their degree of freedom of the was 107. The estimated sample size would be 62 to 158. For the six mediators in the within-CN network model, the degree of freedom was 38. The estimated sample size would be 73 to 282. The number of mediators in the within-DMN network model was 30; the degree of freedom was 530. The estimated sample size would be 18 to 67. The current sample size of 83 could achieve an adequate fit for between-network, within-SN, within-DAN and within-DMN models, and an acceptable fit for the within-CN network.

# Supplementary Results

## Regression: Age-speed and Age-EC associations

Reaction Time measures and Processing Speed indices were summarised in Supplementary Table 1. The latent age-speed regression (Supplementary Table 1, Supplementary Figure 1) indicated that there was significant age-related decline in processing speed (β=-0.535, 95%CI=[-0.700, -0.362]). All PS indices were significantly loaded on the latent speed variable (PS_AudCom_: β=0.602, 95%CI=[0.271, 0.881]; PS_AudInc_: β=0.583, 95%CI= [0.298, 0.868]; PS_VisCom_: β=0.737, 95%CI=[0.404, 0.894] & PS_VisInc_: β=0.827, 95%CI=[0.517, 0.987]).

*Supplementary Table 1 Descriptive statistics of the final sample*

|  | **Young**  **(age:18-28)** | **Middle Age**  **(age:45-55)** | **Older**  **(age:65-75)** |
| --- | --- | --- | --- |
| N | 34 | 25 | 22 |
| Age | 21.6±2.0 | 51.2±3.6 | 67.1±2.9 |
| **Simple RT (ms)** |  |  |  |
| Audial | 231.9±65.5 | 262.5±80.7 | 262.7±106.5 |
| Visual | 244.6±45.1 | 244.5±52.2 | 282.0±90.7 |
| **Choice Reaction Time (ms)** |  |  |  |
| Audial Compatible | 363.8±84.1 | 432.1±106.7 | 472.1±81.8 |
| Audial Incompatible | 399.0±95.5 | 505.2±94.5 | 519.3±94.0 |
| Visual Compatible | 378.1±46.0 | 428.7±80.3 | 470.1±72.8 |
| Visual Incompatible | 440.6±60.1 | 539.5±95.4 | 562.1±102.5 |
| **Processing Speed (ms)** |  |  |  |
| Audial Compatible | 131.9±80.3 | 169.5±122.4 | 209.5±88.8 |
| Audial Incompatible | 167.1±93.1 | 242.6±88.3 | 256.6±88.6 |
| Visual Compatible | 133.5±51.0 | 184.2±68.9 | 188.1±61.2 |
| Visual Incompatible | 196.0±64.0 | 295.1±94.6 | 280.1±74.7 |

Note: Mean and standard deviations raw RT and derived measures were reported here. Processing speed were defined as the RTs of the response mapping tasks. Processing speeds were calculated by subtracting the perceptual-modality-matched simple RTs from the choice RTs. The reported processing speeds were transformed to negative standard score to indicate higher score-better performance association in subsequent analysis.

*Supplementary Figure 1 Latent age-speed regression.*

Above: the model and parameter estimates. Below: scatter plot between latent processing speed score and age. Speed indices and age were transformed into standard score before model fitting.

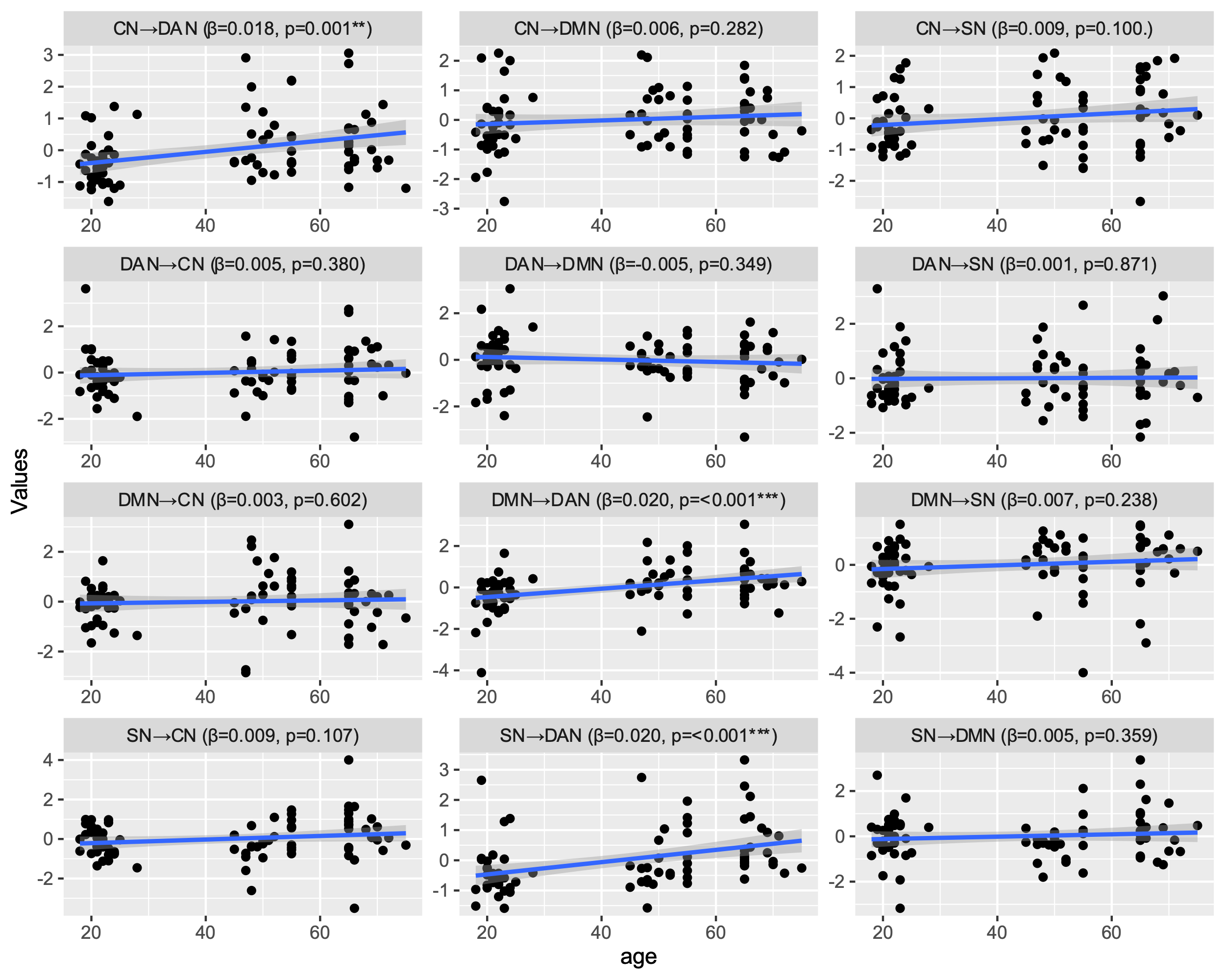


*Supplementary Figure 2 Regressions of network-level connectivity mediators on age.*

(Upper) In each cell, the upper and bottom line of the box indicated upper and lower limit of 95% confidence interval and the middle line bootstrapped mean. Crosses indicated that the confidence interval contained 0. Blue and red shades indicated positive and negative associations respectively. (Lower) Regression plot of effective connectivity measures on age. SN→DAN, CN→DAN and DMN→DAN were significantly positively associated with age. CN: Cerebellar Network, DAN: Dorsal Attention Network; SN: Salient Network; DMN: Default Mode Network

For inter-network age-EC regressions, only influence from SN, CN, DMN to DAN were significantly positively associated with age (SN→DAN: β=0.403, 95%CI=[0.209, 0.567]; CN→DAN: β=0.346, 95%CI=[0.171 0.507]; DMN→DAN: β=0.398, 95%CI=[0.236, 0.538], Figure 4 and Supplementary Table 1). For regional inter-network regressions, a similar pattern was observed between network EC pathways (Figure 5 and Supplementary Table 1). The majority of those SN→DAN (16 out of 16), CN→DAN (9/12) and DMN→DAN (20/24) pathways were significantly positively associated with age, while a minority of SN→CN (6/12), SN→DMN (6/24), DAN→SN (0/16), DAN→CN (3/12), DAN→DMN (0/24), CN→SN (4/12), CN→DMN (6/18), DMN→SN (9/24) and DMN→CN (3/24) pathways were significantly positively associated with age, except one of the DAN→DMN pathways significantly decreased with age. The results indicated that between network effective connectivities increased with age.

*Supplementary Figure 3 Regressions of region-level connectivity mediators on age.*

In each cell, the upper and bottom line of the box indicated upper and lower limit of 95% confidence interval and the middle line bootstrapped mean. Crosses indicated that the confidence interval contained 0. Blue and red shades indicated positive and negative associations respectively. Submatrices circumscribed in orange boxes were within-network connections. Inter-network regional EC (in black boxes) were excluded from further analysis.

*Supplementary Table 2 Regression Analysis on Age-EC Associations*

| **Effective Pathway** | **b** | **95%CI** |  | **Effective Pathway** | **b** | **95%CI** |  | **Effective Pathway** | **b** | **95%CI** |  | **Effective Pathway** | **b** | **95%CI** |
| --- | --- | --- | --- | --- | --- | --- | --- | --- | --- | --- | --- | --- | --- | --- |
| *Systemic EC: Beteen Network* | | |  | *Regional EC: Cerebellar to Dorsal Attention* | | |  | *Regional EC: Default Mode to Cerebellar* | | |  | *Regional EC: Salience to Default Mode* | | |
| DAN→CN | 0.106 | [-0.131, 0.343] |  | **MCV6→RIPS**** | **0.35** | **[0.178, 0.516]** |  | **RFP→LCH6**** | **0.214** | **[0.020, 0.393]** |  | RAI→LFP | 0.188 | [-0.024, 0.329] |
| DAN→DMN | -0.104 | [-0.325, 0.123] |  | **RCH6→LFEF**** | **0.306** | **[0.117, 0.484]** |  | RFP→MCV6 | 0.093 | [-0.115, 0.291] |  | **RAI→LPCC**** | **0.225** | **[0.031, 0.406]** |
| DAN→SN | 0.018 | [-0.203, 0.243] |  | **RCH6→LIPS**** | **0.262** | **[0.060, 0.452]** |  | RFP→RCH6 | 0.026 | [-0.155, 0.209] |  | **RAI→RAG**** | **0.279** | **[0.086, 0.436]** |
| **CN→DAN**** | **0.346** | **[0.171, 0.507]** |  | **RCH6→RFEF**** | **0.257** | **[0.062, 0.432]** |  | RPCC→LCH6 | 0.034 | [-0.160, 0.243] |  | RAI→RFP | 0.179 | [-0.034, 0.329] |
| CN→DMN | 0.119 | [-0.102, 0.334] |  | **RCH6→RIPS**** | **0.295** | **[0.092, 0.485]** |  | RPCC→MCV6 | -0.131 | [-0.319, 0.078] |  | RAI→RPCC | 0.158 | [-0.072, 0.375] |
| CN→SN | 0.184 | [-0.021, 0.392] |  | *Regional EC: Cerebellar to Default Mode* | | |  | RPCC→RCH6 | -0.018 | [-0.212, 0.197] |  | RACC→LAG | -0.059 | [-0.280, 0.165] |
| **DMN→DAN**** | **0.398** | **[0.236, 0.538]** |  | LCH6→LAG | 0.145 | [-0.015, 0.308] |  | *Regional EC: Default Mode to Salience* | | |  | RACC→LFP | -0.148 | [-0.375, 0.094] |
| DMN→CN | 0.059 | [-0.125, 0.243] |  | LCH6→LFP | -0.05 | [-0.254, 0.160] |  | LAG→LAI | 0.086 | [-0.117, 0.299] |  | RACC→LPCC | 0.022 | [-0.194, 0.248] |
| DMN→SN | 0.138 | [-0.068, 0.358] |  | LCH6→LPCC | 0.043 | [-0.168, 0.267] |  | **LAG→LACC**** | **0.371** | **[0.210, 0.516]** |  | RACC→RAG | 0.007 | [-0.221, 0.236] |
| **SN→DAN**** | **0.403** | **[0.209, 0.567]** |  | LCH6→RAG | 0.096 | [-0.109, 0.304] |  | LAG→RAI | 0.142 | [-0.078, 0.358] |  | RACC→RFP | -0.093 | [-0.326, 0.148] |
| SN→CN | 0.183 | [-0.033, 0.387] |  | LCH6→RFP | -0.086 | [-0.305, 0.128] |  | **LAG→RACC**** | **0.358** | **[0.181, 0.521]** |  | RACC→RPCC | -0.016 | [-0.232, 0.214] |
| SN→DMN | 0.102 | [-0.133, 0.310] |  | LCH6→RPCC | 0.009 | [-0.202, 0.217] |  | LFP→LAI | 0.022 | [-0.186, 0.240] |  | *Regional EC: Within Dorsal Attention* | | |
| *Regional EC: Dorsal Attention to Cerebellar* | | |  | **MCV6→LAG**** | **0.249** | **[0.085, 0.398]** |  | **LFP→LACC**** | **0.39** | **[0.213, 0.544]** |  | LFEF→LIPS | 0.118 | [-0.081, 0.316] |
| LFEF→LCH6 | 0.084 | [-0.140, 0.310] |  | MCV6→LFP | -0.108 | [-0.322, 0.117] |  | LFP→RAI | 0.053 | [-0.163, 0.277] |  | LFEF→RFEF | 0.158 | [-0.049, 0.354] |
| LFEF→MCV6 | -0.124 | [-0.338, 0.106] |  | MCV6→LPCC | 0.113 | [-0.118, 0.345] |  | **LFP→RACC**** | **0.338** | **[0.125, 0.521]** |  | LFEF→RIPS | 0.024 | [-0.192, 0.231] |
| LFEF→RCH6 | 0.018 | [-0.225, 0.268] |  | **MCV6→RAG**** | **0.254** | **[0.050, 0.430]** |  | LPCC→LAI | 0.041 | [-0.157, 0.246] |  | **LIPS→LFEF**** | **0.298** | **[0.078, 0.501]** |
| **LIPS→LCH6**** | **0.214** | **[0.019, 0.397]** |  | MCV6→RFP | -0.108 | [-0.322, 0.108] |  | **LPCC→LACC**** | **0.214** | **[0.012, 0.415]** |  | **LIPS→RFEF**** | **0.245** | **[0.030, 0.441]** |
| LIPS→MCV6 | 0.045 | [-0.178, 0.264] |  | MCV6→RPCC | 0.167 | [-0.060, 0.394] |  | LPCC→RAI | 0.157 | [-0.039, 0.350] |  | LIPS→RIPS | 0.096 | [-0.126, 0.315] |
| LIPS→RCH6 | 0.029 | [-0.207, 0.271] |  | **RCH6→LAG**** | **0.306** | **[0.105, 0.487]** |  | **LPCC→RACC**** | **0.242** | **[0.042, 0.437]** |  | **RFEF→LFEF**** | **0.285** | **[0.083, 0.467]** |
| **RFEF→LCH6**** | **0.22** | **[0.027, 0.393]** |  | RCH6→LFP | 0.138 | [-0.072, 0.325] |  | RAG→LAI | -0.009 | [-0.194, 0.218] |  | RFEF→LIPS | 0.194 | [-0.020, 0.397] |
| RFEF→MCV6 | 0.033 | [-0.185, 0.249] |  | **RCH6→LPCC**** | **0.258** | **[0.062, 0.435]** |  | RAG→LACC | 0.144 | [-0.042, 0.339] |  | RFEF→RIPS | 0.087 | [-0.134, 0.303] |
| RFEF→RCH6 | 0.155 | [-0.079, 0.370] |  | **RCH6→RAG**** | **0.283** | **[0.087, 0.465]** |  | RAG→RAI | 0.011 | [-0.189, 0.232] |  | **RIPS→LFEF**** | **0.265** | **[0.062, 0.451]** |
| **RIPS→LCH6**** | **0.303** | **[0.119, 0.471]** |  | RCH6→RFP | 0.091 | [-0.130, 0.297] |  | RAG→RACC | 0.161 | [-0.036, 0.356] |  | RIPS→LIPS | 0.159 | [-0.065, 0.352] |
| RIPS→MCV6 | 0.121 | [-0.109, 0.351] |  | **RCH6→RPCC**** | **0.335** | **[0.153, 0.498]** |  | RFP→LAI | -0.028 | [-0.220, 0.193] |  | **RIPS→RFEF**** | **0.273** | **[0.076, 0.450]** |
| RIPS→RCH6 | 0.189 | [-0.030, 0.398] |  | *Regional EC: Cerebellar to Salience* | | |  | **RFP→LACC**** | **0.322** | **[0.116, 0.499]** |  | *Regional EC: Within Cerebellar* | | |
| *Regional EC: Dorsal Attention to Default Mode* | | |  | LCH6→LAI | 0.131 | [-0.081, 0.333] |  | RFP→RAI | -0.02 | [-0.224, 0.219] |  | LCH6→MCV6 | 0.079 | [-0.116, 0.268] |
| **LFEF→LAG**** | **-0.218** | **[-0.397, -0.016]** |  | LCH6→LACC | 0.17 | [-0.053, 0.393] |  | RFP→RACC | 0.228 | [-0.016, 0.444] |  | LCH6→RCH6 | 0.083 | [-0.148, 0.299] |
| LFEF→LFP | -0.089 | [-0.314, 0.141] |  | LCH6→RAI | -0.044 | [-0.256, 0.172] |  | RPCC→LAI | -0.02 | [-0.212, 0.186] |  | **MCV6→LCH6**** | **0.243** | **[0.067, 0.415]** |
| LFEF→LPCC | -0.044 | [-0.256, 0.180] |  | LCH6→RACC | 0.136 | [-0.077, 0.337] |  | **RPCC→LACC**** | **0.235** | **[0.045, 0.431]** |  | MCV6→RCH6 | 0.183 | [-0.036, 0.385] |
| LFEF→RAG | -0.131 | [-0.326, 0.085] |  | MCV6→LAI | 0.07 | [-0.146, 0.275] |  | RPCC→RAI | 0.073 | [-0.127, 0.282] |  | **RCH6→LCH6**** | **0.401** | **[0.227, 0.560]** |
| LFEF→RFP | -0.038 | [-0.260, 0.175] |  | **MCV6→LACC**** | **0.217** | **[0.025, 0.396]** |  | **RPCC→RACC**** | **0.242** | **[0.028, 0.445]** |  | **RCH6→MCV6**** | **0.358** | **[0.175, 0.520]** |
| LFEF→RPCC | -0.061 | [-0.278, 0.163] |  | MCV6→RAI | -0.015 | [-0.217, 0.186] |  | *Regional EC: Salience to Dorsal Attention* | | |  | *Regional EC: Within Default Mode* | | |
| LIPS→LAG | -0.121 | [-0.312, 0.062] |  | **MCV6→RACC**** | **0.247** | **[0.050, 0.435]** |  | **LAI→LFEF**** | **0.415** | **[0.278, 0.544]** |  | **LAG→LFP**** | **-0.235** | **[-0.413, -0.046]** |
| LIPS→LFP | -0.007 | [-0.211, 0.192] |  | RCH6→LAI | 0.19 | [-0.027, 0.388] |  | **LAI→LIPS**** | **0.449** | **[0.312, 0.577]** |  | LAG→LPCC | 0.014 | [-0.214, 0.240] |
| LIPS→LPCC | -0.028 | [-0.246, 0.199] |  | **RCH6→LACC**** | **0.309** | **[0.099, 0.497]** |  | **LAI→RFEF**** | **0.332** | **[0.179, 0.480]** |  | LAG→RAG | -0.016 | [-0.236, 0.203] |
| LIPS→RAG | -0.043 | [-0.244, 0.169] |  | RCH6→RAI | 0.075 | [-0.144, 0.291] |  | **LAI→RIPS**** | **0.377** | **[0.193, 0.547]** |  | **LAG→RFP**** | **-0.263** | **[-0.438, -0.069]** |
| LIPS→RFP | 0.016 | [-0.184, 0.209] |  | **RCH6→RACC**** | **0.306** | **[0.109, 0.478]** |  | **LACC→LFEF**** | **0.385** | **[0.228, 0.541]** |  | LAG→RPCC | 0.017 | [-0.209, 0.246] |
| LIPS→RPCC | -0.083 | [-0.300, 0.137] |  | *Regional EC: Default Mode to Dorsal Attention* | | |  | **LACC→LIPS**** | **0.33** | **[0.139, 0.517]** |  | LFP→LAG | 0.065 | [-0.166, 0.286] |
| RFEF→LAG | -0.141 | [-0.351, 0.089] |  | **LAG→LFEF**** | **0.457** | **[0.339, 0.563]** |  | **LACC→RFEF**** | **0.35** | **[0.179, 0.506]** |  | LFP→LPCC | -0.042 | [-0.261, 0.177] |
| RFEF→LFP | -0.029 | [-0.263, 0.201] |  | **LAG→LIPS**** | **0.291** | **[0.148, 0.434]** |  | **LACC→RIPS**** | **0.257** | **[0.067, 0.436]** |  | LFP→RAG | 0.136 | [-0.073, 0.335] |
| RFEF→LPCC | 0.065 | [-0.163, 0.287] |  | **LAG→RFEF**** | **0.401** | **[0.261, 0.526]** |  | **RAI→LFEF**** | **0.389** | **[0.244, 0.520]** |  | **LFP→RFP**** | **-0.383** | **[-0.533, -0.208]** |
| RFEF→RAG | -0.118 | [-0.335, 0.120] |  | **LAG→RIPS**** | **0.372** | **[0.193, 0.530]** |  | **RAI→LIPS**** | **0.41** | **[0.261, 0.538]** |  | LFP→RPCC | -0.026 | [-0.238, 0.199] |
| RFEF→RFP | -0.003 | [-0.226, 0.219] |  | **LFP→LFEF**** | **0.413** | **[0.243, 0.558]** |  | **RAI→RFEF**** | **0.28** | **[0.103, 0.433]** |  | **LPCC→LAG**** | **-0.185** | **[-0.353, -0.001]** |
| RFEF→RPCC | 0.025 | [-0.203, 0.260] |  | **LFP→LIPS**** | **0.393** | **[0.238, 0.530]** |  | **RAI→RIPS**** | **0.326** | **[0.138, 0.485]** |  | **LPCC→LFP**** | **-0.307** | **[-0.462, -0.127]** |
| RIPS→LAG | -0.141 | [-0.338, 0.072] |  | **LFP→RFEF**** | **0.375** | **[0.192, 0.531]** |  | **RACC→LFEF**** | **0.334** | **[0.133, 0.516]** |  | LPCC→RAG | -0.076 | [-0.253, 0.127] |
| RIPS→LFP | -0.018 | [-0.244, 0.201] |  | **LFP→RIPS**** | **0.364** | **[0.197, 0.512]** |  | **RACC→LIPS**** | **0.239** | **[0.011, 0.453]** |  | **LPCC→RFP**** | **-0.285** | **[-0.457, -0.088]** |
| RIPS→LPCC | 0.099 | [-0.132, 0.327] |  | **LPCC→LFEF**** | **0.251** | **[0.063, 0.430]** |  | **RACC→RFEF**** | **0.322** | **[0.118, 0.508]** |  | LPCC→RPCC | -0.119 | [-0.318, 0.090] |
| RIPS→RAG | -0.088 | [-0.276, 0.120] |  | LPCC→LIPS | 0.112 | [-0.088, 0.305] |  | **RACC→RIPS**** | **0.261** | **[0.023, 0.490]** |  | **RAG→LAG**** | **-0.227** | **[-0.406, -0.033]** |
| RIPS→RFP | -0.031 | [-0.242, 0.186] |  | **LPCC→RFEF**** | **0.271** | **[0.071, 0.463]** |  | *Regional EC: Salience to Cerebellar* | | |  | **RAG→LFP**** | **-0.305** | **[-0.457, -0.136]** |
| RIPS→RPCC | 0.039 | [-0.187, 0.275] |  | LPCC→RIPS | 0.161 | [-0.086, 0.399] |  | **LAI→LCH6**** | **0.406** | **[0.184, 0.598]** |  | RAG→LPCC | -0.182 | [-0.357, 0.009] |
| *Regional EC: Dorsal Attention to Salience* | | |  | **RAG→LFEF**** | **0.367** | **[0.192, 0.530]** |  | **LAI→MCV6**** | **0.267** | **[0.085, 0.441]** |  | **RAG→RFP**** | **-0.27** | **[-0.431, -0.082]** |
| LFEF→LAI | -0.094 | [-0.307, 0.112] |  | **RAG→LIPS**** | **0.202** | **[0.010, 0.382]** |  | **LAI→RCH6**** | **0.388** | **[0.225, 0.532]** |  | RAG→RPCC | -0.128 | [-0.325, 0.083] |
| LFEF→LACC | -0.028 | [-0.235, 0.177] |  | **RAG→RFEF**** | **0.334** | **[0.145, 0.506]** |  | LACC→LCH6 | 0.173 | [-0.043, 0.380] |  | RFP→LAG | -0.041 | [-0.266, 0.173] |
| LFEF→RAI | -0.121 | [-0.305, 0.103] |  | **RAG→RIPS**** | **0.295** | **[0.099, 0.470]** |  | LACC→MCV6 | -0.008 | [-0.237, 0.210] |  | **RFP→LFP**** | **-0.388** | **[-0.545, -0.211]** |
| LFEF→RACC | -0.049 | [-0.256, 0.179] |  | **RFP→LFEF**** | **0.323** | **[0.108, 0.505]** |  | LACC→RCH6 | 0.107 | [-0.125, 0.323] |  | RFP→LPCC | -0.12 | [-0.331, 0.086] |
| LIPS→LAI | 0.007 | [-0.209, 0.219] |  | **RFP→LIPS**** | **0.329** | **[0.128, 0.505]** |  | **RAI→LCH6**** | **0.357** | **[0.144, 0.543]** |  | RFP→RAG | 0.06 | [-0.159, 0.282] |
| LIPS→LACC* | 0.186 | [-0.012, 0.389] |  | **RFP→RFEF**** | **0.345** | **[0.162, 0.499]** |  | **RAI→MCV6**** | **0.218** | **[0.007, 0.412]** |  | RFP→RPCC | -0.058 | [-0.279, 0.153] |
| LIPS→RAI | -0.067 | [-0.270, 0.160] |  | **RFP→RIPS**** | **0.252** | **[0.076, 0.406]** |  | **RAI→RCH6**** | **0.284** | **[0.076, 0.470]** |  | **RPCC→LAG**** | **-0.234** | **[-0.416, -0.036]** |
| LIPS→RACC | 0.132 | [-0.106, 0.355] |  | **RPCC→LFEF**** | **0.300** | **[0.124, 0.464]** |  | RACC→LCH6 | 0.188 | [-0.036, 0.383] |  | **RPCC→LFP**** | **-0.352** | **[-0.507, -0.170]** |
| RFEF→LAI | -0.047 | [-0.268, 0.164] |  | RPCC→LIPS | 0.156 | [-0.038, 0.342] |  | RACC→MCV6 | -0.069 | [-0.291, 0.153] |  | RPCC→LPCC | -0.192 | [-0.398, 0.009] |
| RFEF→LACC | 0.113 | [-0.098, 0.312] |  | **RPCC→RFEF**** | **0.326** | **[0.135, 0.506]** |  | RACC→RCH6 | 0.085 | [-0.168, 0.324] |  | RPCC→RAG | -0.095 | [-0.281, 0.104] |
| RFEF→RAI | -0.114 | [-0.318, 0.113] |  | RPCC→RIPS | 0.141 | [-0.084, 0.360] |  | *Regional EC: Salience to Default Mode* | | |  | **RPCC→RFP**** | **-0.291** | **[-0.469, -0.086]** |
| RFEF→RACC | 0.03 | [-0.204, 0.250] |  | *Regional EC: Default Mode to Cerebellar* | | |  | **LAI→LAG**** | **0.276** | **[0.091, 0.442]** |  | *Regional EC: Within Salience* | | |
| RIPS→LAI | 0.081 | [-0.160, 0.307] |  | **LAG→LCH6**** | **0.188** | **[0.010, 0.355]** |  | LAI→LFP | 0.19 | [-0.026, 0.375] |  | **LAI→LACC**** | **0.337** | **[0.172, 0.490]** |
| RIPS→LACC* | 0.204 | [-0.016, 0.405] |  | LAG→MCV6 | 0.074 | [-0.110, 0.246] |  | **LAI→LPCC**** | **0.259** | **[0.069, 0.446]** |  | LAI→RAI | -0.027 | [-0.245, 0.186] |
| RIPS→RAI | -0.044 | [-0.282, 0.188] |  | LAG→RCH6 | 0.045 | [-0.147, 0.221] |  | **LAI→RAG**** | **0.238** | **[0.027, 0.420]** |  | **LAI→RACC**** | **0.342** | **[0.169, 0.498]** |
| RIPS→RACC | 0.165 | [-0.070, 0.363] |  | **LFP→LCH6**** | **0.242** | **[0.046, 0.421]** |  | LAI→RFP | 0.143 | [-0.081, 0.337] |  | LACC→LAI | 0.001 | [-0.216, 0.226] |
| *Regional EC: Cerebellar to Dorsal Attention* | | |  | LFP→MCV6 | 0.1 | [-0.112, 0.296] |  | LAI→RPCC | 0.164 | [-0.070, 0.387] |  | LACC→RAI | -0.087 | [-0.297, 0.152] |
| **LCH6→LFEF**** | **0.255** | **[0.049, 0.443]** |  | LFP→RCH6 | 0.053 | [-0.129, 0.244] |  | LACC→LAG | -0.124 | [-0.316, 0.086] |  | LACC→RACC | 0.13 | [-0.098, 0.349] |
| LCH6→LIPS | 0.158 | [-0.023, 0.341] |  | LPCC→LCH6 | 0.138 | [-0.063, 0.344] |  | LACC→LFP | -0.151 | [-0.382, 0.087] |  | RAI→LAI | -0.194 | [-0.419, 0.021] |
| LCH6→RFEF | 0.15 | [-0.063, 0.349] |  | LPCC→MCV6 | -0.038 | [-0.222, 0.164] |  | LACC→LPCC | -0.093 | [-0.290, 0.113] |  | **RAI→LACC**** | **0.291** | **[0.116, 0.438]** |
| LCH6→RIPS | 0.19 | [-0.033, 0.414] |  | LPCC→RCH6 | 0.065 | [-0.113, 0.249] |  | LACC→RAG | -0.015 | [-0.218, 0.191] |  | **RAI→RACC**** | **0.244** | **[0.058, 0.410]** |
| **MCV6→LFEF**** | **0.32** | **[0.156, 0.475]** |  | RAG→LCH6 | 0.038 | [-0.169, 0.244] |  | LACC→RFP | -0.155 | [-0.371, 0.076] |  | RACC→LAI | -0.077 | [-0.289, 0.154] |
| **MCV6→LIPS**** | **0.368** | **[0.221, 0.523]** |  | RAG→MCV6 | -0.055 | [-0.260, 0.174] |  | LACC→RPCC | -0.118 | [-0.320, 0.097] |  | RACC→LACC | 0.107 | [-0.125, 0.348] |
| **MCV6→RFEF**** | **0.285** | **[0.110, 0.442]** |  | RAG→RCH6 | -0.021 | [-0.239, 0.198] |  | RAI→LAG** | 0.249 | **[0.081, 0.382]** |  | RACC→RAI | -0.152 | [-0.356, 0.079] |

*Supplementary Table 3* Inter-Network Parallel Mediation Model

| **Parameter** | **Formula** | **𝛽** | **95%CI** |
| --- | --- | --- | --- |
| Latent Speed | PS=~PS_AudCom_ | 0.654 | **[0.371, 0.900]** |
|  | PS=~PS_AudInc_ | 0.566 | **[0.309, 0.817]** |
|  | PS=~PS_VisCom_ | 0.599 | **[0.380, 0.804]** |
|  | PS=~PS_VisInc_ | 0.782 | **[0.537, 1.088]** |
| a-paths | SN→AN~Age | 0.403 | **[0.208, 0.567]** |
|  | CN→AN~Age | 0.346 | **[0.171, 0.507]** |
|  | DN→AN~Age | 0.398 | **[0.236, 0.538]** |
| b-paths | PS~SN→AN | 0.355 | **[0.025, 0.626]** |
|  | PS~CN→AN | -0.445 | **[-0.759, -0.090]** |
|  | PS~DN→AN | 0.066 | [-0.216, 0.321] |
| c-path | C | -0.618 | **[-0.896, -0.334]** |
| Indirect Effects | SN→AN | 0.139 | **[0.011, 0.269]** |
|  | CN→AN | -0.154 | **[-0.300, -0.025]** |
|  | DN→AN | 0.024 | [-0.096, 0.130] |
| Total Effect | Total | -0.609 | **[-0.833, -0.356]** |
| Covariance | CN→AN~~SN→AN | 0.594 | **[0.372, 0.757]** |
|  | PS_AudCom_~~PS_AudInc_ | 0.464 | **[0.043, 0.700]** |
|  | PS_VisCom_~~PS_VisInc_ | 0.51 | [-0.320, 0.809] |
|  | PS_AudCom_~~PS_VisInc_ | -0.293 | [-1.481, 0.129] |
|  | PS_AudInc_~~PS_VisCom_ | -0.035 | [-0.301, 0.187] |
| Variance | PS_AudCom_~~PS_AudCom_ | 0.554 | **[0.190, 0.862]** |
|  | PS_AudInc_~~PS_AudInc_ | 0.663 | **[0.333, 0.905]** |
|  | PS_VisCom_~~PS_VisCom_ | 0.63 | **[0.354, 0.855]** |
|  | PS_VisInc_~~PS_VisInc_ | 0.368 | [-0.184, 0.711] |
|  | SN→AN~~SN→AN | 0.829 | **[0.678, 0.957]** |
|  | CN→AN~~CN→AN | 0.873 | **[0.743, 0.971]** |
|  | DN→AN~~DN→AN | 0.836 | **[0.710, 0.944]** |
|  | PS~~PS | 0.449 | **[0.120, 0.711]** |
|  | Age~~Age | 1 | **[1.000, 1.000]** |

Supplementary Table 4 Intra-Salience Network Parallel Mediation Model

| **Parameter** | **Formula** | **𝛽** | **95%CI** |
| --- | --- | --- | --- |
| Latent Speed | PS=~PS_AudCom_ | 0.66 | **[0.362, 0.952]** |
|  | PS=~PS_AudInc_ | 0.648 | **[0.376, 0.929]** |
|  | PS=~PS_VisCom_ | 0.565 | **[0.281, 0.818]** |
|  | PS=~PS_VisInc_ | 0.68 | **[0.449, 0.910]** |
| a-paths | LAI→LMFC~Age | 0.337 | **[0.172, 0.490]** |
|  | RAI→LMFC~Age | 0.291 | **[0.116, 0.438]** |
|  | LAI→RMFC~Age | 0.342 | **[0.168, 0.499]** |
|  | RAI→RMFC~Age | 0.244 | **[0.058, 0.410]** |
| b-paths | PS~LAI→LMFC | 0.095 | [-0.749, 0.934] |
|  | PS~RAI→LMFC | -0.222 | [-1.040, 0.598] |
|  | PS~LAI→RMFC | -0.281 | [-0.816, 0.350] |
|  | PS~RAI→RMFC | 0.373 | [-0.155, 0.944] |
| Indirect Effect | LAI→LMFC | 0.037 | [-0.253, 0.352] |
|  | RAI→LMFC | -0.071 | [-0.348, 0.161] |
|  | LAI→RMFC | -0.094 | [-0.308, 0.133] |
|  | RAI→RMFC | 0.091 | [-0.041, 0.274] |
| c-path |  | -0.614 | **[-0.862, -0.366]** |
| Total Effect |  | -0.652 | **[-0.861, -0.437]** |
| Covariance | LAI→LMFC~~LAI→RMFC | 0.777 | **[0.627, 0.880]** |
|  | LAI→LMFC~~RAI→LMFC | 0.779 | **[0.681, 0.851]** |
|  | RAI→LMFC~~RAI→RMFC | 0.757 | **[0.592, 0.873]** |
|  | LAI→RMFC~~RAI→RMFC | 0.729 | **[0.569, 0.852]** |
|  | LAI→LMFC~~RAI→RMFC | 0.631 | **[0.433, 0.781]** |
|  | PS_VisCom_~~PS_VisInc_ | 0.563 | **[0.190, 0.771]** |
|  | PS_AudCom_~~PS_AudInc_ | 0.307 | [-1.083, 0.686] |
|  | RAI→LMFC~~LAI→RMFC | 0.571 | **[0.345, 0.743]** |
|  | PS_AudCom_~~PS_VisInc_ | -0.081 | [-0.545, 0.207] |
|  | PS_AudInc_~~PS_VisCom_ | -0.107 | [-0.500, 0.138] |
|  | PS_AudCom_~~PS_AudCom_ | 0.533 | **[0.093, 0.869]** |
| Variance | PS_AudInc_~~PS_AudInc_ | 0.544 | **[0.138, 0.858]** |
|  | PS_VisCom_~~PS_VisCom_ | 0.661 | **[0.331, 0.921]** |
|  | PS_VisInc_~~PS_VisInc_ | 0.52 | **[0.172, 0.798]** |
|  | LAI→LMFC~~LAI→LMFC | 0.88 | **[0.760, 0.970]** |
|  | RAI→LMFC~~RAI→LMFC | 0.909 | **[0.808, 0.987]** |
|  | LAI→RMFC~~LAI→RMFC | 0.876 | **[0.751, 0.972]** |
|  | RAI→RMFC~~RAI→RMFC | 0.932 | **[0.832, 0.996]** |
|  | PS~~PS | 0.444 | **[0.104, 0.715]** |
|  | Age~~Age | 1 | **[1.000, 1.000]** |

Supplementary Table 5 Intra-Cerebellar Network Parallel Mediation Model

| **Parameter** | **Formula** | **𝛽** | **95%CI** |
| --- | --- | --- | --- |
| Latent Speed | PS=~PS_AudCom_ | 0.67 | **[0.380, 0.965]** |
|  | PS=~PS_AudInc_ | 0.718 | **[0.404, 1.106]** |
|  | PS=~PS_VisCom_ | 0.592 | **[0.339, 0.841]** |
|  | PS=~PS_VisInc_ | 0.722 | **[0.450, 1.068]** |
| a-paths | MCV6→LCH6~Age | 0.243 | **[0.067, 0.415]** |
|  | RCH6→LCH6~Age | 0.407 | **[0.230, 0.568]** |
|  | RCH6→MCV6~Age | 0.358 | **[0.175, 0.520]** |
| b-paths | PS~MCV6→LCH6 | -0.086 | [-0.410, 0.237] |
|  | PS~RCH6→LCH6 | -0.24 | [-0.824, 0.290] |
|  | PS~RCH6→MCV6 | 0.08 | [-0.408, 0.608] |
| Indirect Effects | MCV6→LCH6 | -0.019 | [-0.110, 0.069] |
|  | RCH6→LCH6 | -0.097 | [-0.355, 0.123] |
|  | RCH6→MCV6 | 0.026 | [-0.166, 0.225] |
| c-path |  | -0.538 | **[-0.815, -0.261]** |
| Total Effect |  | -0.629 | **[-0.893, -0.362]** |
| Covariance | RCH6→LCH6~~RCH6→MCV6 | 0.772 | **[0.660, 0.862]** |
|  | MCV6→LCH6~~RCH6→LCH6 | 0.365 | **[0.221, 0.493]** |
|  | PS_VisCom_~~PS_VisInc_ | 0.536 | [-0.109, 0.856] |
|  | PS_AudCom_~~PS_AudInc_ | 0.164 | [-2.101, 0.682] |
|  | PS_AudInc_~~PS_VisCom_ | -0.339 | [-1.737, 0.150] |
|  | PS_AudCom_~~PS_VisInc_ | -0.266 | [-1.833, 0.203] |
|  | PS_AudInc_~~PS_VisInc_ | -0.617 | [-4.371, 0.284] |
| Variance | PS_AudCom_~~PS_AudCom_ | 0.521 | **[0.068, 0.855]** |
|  | PS_AudInc_~~PS_AudInc_ | 0.441 | [-0.223, 0.837] |
|  | PS_VisCom_~~PS_VisCom_ | 0.634 | **[0.292, 0.885]** |
|  | PS_VisInc_~~PS_VisInc_ | 0.451 | [-0.142, 0.797] |
|  | MCV6→LCH6~~MCV6→LCH6 | 0.933 | **[0.827, 0.995]** |
|  | RCH6→LCH6~~RCH6→LCH6 | 0.826 | **[0.678, 0.947]** |
|  | RCH6→MCV6~~RCH6→MCV6 | 0.864 | **[0.729, 0.970]** |
|  | PS~~PS | 0.498 | **[0.079, 0.801]** |
|  | Age~~Age | 1 | **[1.000, 1.000]** |

Supplementary Table 6 Intra-Dorsal Attention Network Parallel Mediation Model

| **Parameter** | **Formula** | **𝛽** | **95%CI** |
| --- | --- | --- | --- |
| Latent Speed | PS=~PS_AudCom_ | 0.702 | **[0.343, 1.107]** |
|  | PS=~PS_AudInc_ | 0.673 | **[0.329, 1.014]** |
|  | PS=~PS_VisCom_ | 0.559 | **[0.265, 0.878]** |
|  | PS=~PS_VisInc_ | 0.679 | **[0.371, 1.093]** |
| a-paths | RFEF→LFEF~Age | 0.285 | **[0.083, 0.467]** |
|  | LIPS→LFEF~Age | 0.298 | **[0.078, 0.502]** |
|  | RIPS→LFEF~Age | 0.265 | **[0.061, 0.451]** |
|  | LIPS→RFEF~Age | 0.245 | **[0.031, 0.441]** |
|  | RIPS→RFEF~Age | 0.273 | **[0.077, 0.449]** |
| b-paths | PS~RFEF→LFEF | -0.092 | [-0.481, 0.290] |
|  | PS~LIPS→LFEF | -0.238 | [-0.905, 0.478] |
|  | PS~RIPS→LFEF | 0.241 | [-0.611, 1.029] |
|  | PS~LIPS→RFEF | 0.224 | [-0.483, 0.912] |
|  | PS~RIPS→RFEF | -0.214 | [-1.026, 0.587] |
| c-path |  | -0.567 | **[-0.862, -0.200]** |
| Indirect Effects | RFEF→LFEF | -0.028 | [-0.170, 0.092] |
|  | LIPS→LFEF | -0.08 | [-0.338, 0.133] |
|  | RIPS→LFEF | 0.068 | [-0.174, 0.345] |
|  | LIPS→RFEF | 0.052 | [-0.141, 0.261] |
|  | RIPS→RFEF | -0.06 | [-0.334, 0.174] |
| Total Effect |  | -0.615 | **[-0.854, -0.263]** |
| Covariance | RIPS→LFEF~~RIPS→RFEF | 0.768 | **[0.675, 0.845]** |
|  | LIPS→RFEF~~RIPS→RFEF | 0.745 | **[0.643, 0.831]** |
|  | LIPS→LFEF~~RIPS→LFEF | 0.722 | **[0.584, 0.826]** |
|  | LIPS→LFEF~~LIPS→RFEF | 0.703 | **[0.542, 0.832]** |
|  | PS_VisCom_~~PS_VisInc_ | 0.535 | [-0.386, 0.797] |
|  | RFEF→LFEF~~RIPS→LFEF | 0.628 | **[0.458, 0.778]** |
|  | LIPS→LFEF~~RIPS→RFEF | 0.596 | **[0.414, 0.740]** |
|  | PS_AudCom_~~PS_AudInc_ | -0.093 | [-2.640, 0.707] |
|  | RFEF→LFEF~~RIPS→RFEF | 0.54 | **[0.319, 0.716]** |
|  | RFEF→LFEF~~LIPS→LFEF | 0.487 | **[0.275, 0.667]** |
|  | RIPS→LFEF~~LIPS→RFEF | 0.463 | **[0.253, 0.651]** |
|  | RFEF_LFEF~~LIPS_RFEF | 0.443 | **[0.242, 0.618]** |
|  | PS_AudCom_~~PS_VisInc_ | -0.129 | [-0.980, 0.266] |
|  | PS_AudInc_~~PS_VisCom_ | -0.135 | [-0.789, 0.164] |
|  | PS_AudCom_~~PS_AudCom_ | 0.458 | [-0.225, 0.882] |
| Variance | PS_AudInc_~~PS_AudInc_ | 0.508 | [-0.029, 0.892] |
|  | PS_VisCom_~~PS_VisCom_ | 0.659 | **[0.229, 0.930]** |
|  | PS_VisInc_~~PS_VisInc_ | 0.476 | [-0.194, 0.862] |
|  | RFEF→LFEF~~RFEF→LFEF | 0.909 | **[0.781, 0.993]** |
|  | LIPS→LFEF~~LIPS→LFEF | 0.899 | **[0.748, 0.994]** |
|  | RIPS→LFEF~~RIPS→LFEF | 0.92 | **[0.797, 0.996]** |
|  | LIPS→RFEF~~LIPS→RFEF | 0.929 | **[0.805, 0.999]** |
|  | RIPS→RFEF~~RIPS→RFEF | 0.917 | **[0.798, 0.994]** |
|  | PS~~PS | 0.502 | **[0.159, 0.834]** |
|  | Age~~Age | 1 | **[1.000, 1.000]** |

Supplementary Table 7 Intra-Default Mode Network Parallel Mediation Model

| **Parameter** | **Formula** | **𝛽** | **95%CI** |  | **Parameter** | **Formula** | **𝛽** | **95%CI** |  |
| --- | --- | --- | --- | --- | --- | --- | --- | --- | --- |
| Latent Speed | PS=~PS_AudCom_ | 0.578 | **[0.212, 1.133]** |  | Covariance | RFP→LFP~~LAG→RFP | 0.584 | **[0.411, 0.726]** |  |
|  | PS=~PS_AudInc_ | 0.602 | **[0.262, 0.994]** |  |  | RAG→LFP~~RAG→LAG | 0.564 | **[0.356, 0.747]** |  |
|  | PS=~PS_VisCom_ | 0.696 | **[0.249, 1.377]** |  |  | RAG→LFP~~RPCC→RFP | 0.554 | **[0.388, 0.688]** |  |
|  | PS=~PS_VisInc_ | 0.783 | **[0.421, 1.365]** |  |  | RAG→RFP~~LPCC→RFP | 0.55 | **[0.352, 0.704]** |  |
| a-paths | RFP→LFP | -0.397 | **[-0.553, -0.218]** |  |  | PS_AudCom_~~PS_AudInc_ | 0.367 | [-1.394, 0.726] |  |
|  | LAG→LFP | -0.238 | **[-0.419, -0.046]** |  |  | LAG→RFP~~RAG→RFP | 0.545 | **[0.333, 0.723]** |  |
|  | RAG→LFP | -0.315 | **[-0.471, -0.137]** |  |  | LAG→LFP~~LFP→RFP | 0.552 | **[0.384, 0.682]** |  |
|  | LPCC→LFP | -0.319 | **[-0.480, -0.130]** |  |  | LPCC→RFP~~LPCC→LAG | 0.486 | **[0.247, 0.706]** |  |
|  | RPCC→LFP | -0.367 | **[-0.523, -0.177]** |  |  | RPCC→RFP~~RPCC→LAG | 0.479 | **[0.332, 0.627]** |  |
|  | LFP→RFP | -0.382 | **[-0.531, -0.207]** |  |  | LFP→RFP~~LPCC→RFP | 0.497 | **[0.308, 0.681]** |  |
|  | LAG→RFP | -0.268 | **[-0.444, -0.069]** |  |  | RPCC→LFP~~RAG→RFP | 0.532 | **[0.389, 0.660]** |  |
|  | RAG→RFP | -0.281 | **[-0.446, -0.085]** |  |  | RAG→LFP~~LPCC→LFP | 0.526 | **[0.360, 0.660]** |  |
|  | LPCC→RFP | -0.299 | **[-0.479, -0.092]** |  |  | LPCC→LFP~~RPCC→LAG | 0.483 | **[0.317, 0.642]** |  |
|  | RPCC→RFP | -0.306 | **[-0.489, -0.091]** |  |  | RPCC→LFP~~LPCC→LAG | 0.441 | **[0.287, 0.586]** |  |
|  | RAG→LAG | -0.228 | **[-0.406, -0.033]** |  |  | RFP→LFP~~LPCC→LFP | 0.461 | **[0.267, 0.639]** |  |
|  | LPCC→LAG | -0.184 | [-0.353, 0.003] |  |  | RFP→LFP~~LPCC→RFP | 0.462 | **[0.291, 0.628]** |  |
|  | RPCC→LAG | -0.236 | **[-0.421, -0.036]** |  |  | LPCC→LFP~~LFP→RFP | 0.434 | **[0.240, 0.616]** |  |
| b-paths | RFP→LFP | -0.128 | [-0.798, 0.558] |  |  | RAG→LFP~~LPCC→RFP | 0.463 | **[0.287, 0.608]** |  |
|  | LAG→LFP | -0.222 | [-1.356, 0.940] |  |  | RAG→LAG~~RPCC→LAG | 0.518 | **[0.348, 0.667]** |  |
|  | RAG→LFP | 0.004 | [-0.912, 1.029] |  |  | LPCC→LFP~~RAG→RFP | 0.474 | **[0.325, 0.598]** |  |
|  | LPCC→LFP | 0.424 | [-1.694, 2.491] |  |  | LPCC→RFP~~RPCC→LAG | 0.42 | **[0.212, 0.613]** |  |
|  | RPCC→LFP | -0.361 | [-2.302, 1.451] |  |  | LAG→LFP~~LPCC→RFP | 0.453 | **[0.266, 0.614]** |  |
|  | LFP→RFP | 0.09 | [-0.710, 0.826] |  |  | LAG→LFP~~RPCC→RFP | 0.456 | **[0.266, 0.625]** |  |
|  | LAG→RFP | 0.103 | [-1.106, 1.314] |  |  | RAG→LFP~~LAG→RFP | 0.419 | **[0.216, 0.616]** |  |
|  | RAG→RFP | 0.217 | [-0.861, 1.161] |  |  | RPCC→RFP~~LPCC→LAG | 0.373 | **[0.206, 0.536]** |  |
|  | LPCC→RFP | -0.543 | [-2.420, 1.493] |  |  | LFP→RFP~~RAG→RFP | 0.426 | **[0.180, 0.652]** |  |
|  | RPCC→RFP | 0.087 | [-1.760, 1.894] |  |  | RAG→RFP~~RAG→LAG | 0.399 | **[0.196, 0.614]** |  |
|  | RAG→LAG | 0.027 | [-0.394, 0.440] |  |  | LAG→LFP~~RAG→LFP | 0.401 | **[0.169, 0.614]** |  |
|  | LPCC→LAG | -0.069 | [-1.161, 1.084] |  |  | RAG→LAG~~LPCC→LAG | 0.439 | **[0.261, 0.600]** |  |
|  | RPCC→LAG | 0.335 | [-0.829, 1.499] |  |  | RFP→LFP~~RAG→RFP | 0.38 | **[0.172, 0.560]** |  |
| Indirect Effect | RFP→LFP | 0.05 | [-0.243, 0.334] |  |  | RFP→LFP~~RAG→LFP | 0.363 | **[0.150, 0.572]** |  |
|  | LAG→LFP | 0.052 | [-0.277, 0.401] |  |  | RFP→LFP~~RPCC→LAG | 0.262 | **[0.127, 0.402]** |  |
|  | RAG→LFP | -0.009 | [-0.377, 0.282] |  |  | RAG→LFP~~RPCC→LAG | 0.351 | **[0.126, 0.549]** |  |
|  | LPCC→LFP | -0.146 | [-0.933, 0.558] |  |  | RPCC→RFP~~RAG→LAG | 0.297 | **[0.163, 0.427]** |  |
|  | RPCC→LFP | 0.135 | [-0.569, 0.931] |  |  | LAG→LFP~~RAG→RFP | 0.357 | **[0.139, 0.543]** |  |
|  | LFP→RFP | -0.038 | [-0.357, 0.277] |  |  | RAG→LFP~~LFP→RFP | 0.31 | **[0.077, 0.544]** |  |
|  | LAG→RFP | -0.031 | [-0.417, 0.321] |  |  | PS_VisCom_~~PS_VisInc_ | 0.106 | [-2.911, 0.767] |  |
|  | RAG→RFP | -0.054 | [-0.359, 0.284] |  |  | LPCC→RFP~~RAG→LAG | 0.27 | **[0.092, 0.441]** |  |
|  | LPCC→RFP | 0.177 | [-0.442, 0.911] |  |  | LAG→RFP~~LPCC→LAG | 0.221 | **[0.066, 0.386]** |  |
|  | RPCC→RFP | -0.029 | [-0.676, 0.602] |  |  | LAG→LFP~~RPCC→LAG | 0.257 | **[0.091, 0.427]** |  |
|  | RAG→LAG | -0.007 | [-0.120, 0.110] |  |  | LAG→LFP~~LPCC→LAG | 0.248 | **[0.077, 0.423]** |  |
|  | LPCC→LAG | 0.012 | [-0.233, 0.266] |  |  | LAG→RFP~~RPCC→LAG | 0.219 | **[0.076, 0.375]** |  |
|  | RPCC→LAG | -0.08 | [-0.425, 0.209] |  |  | RAG→LFP~~LPCC→LAG | 0.277 | **[0.055, 0.482]** |  |
|  | c | -0.605 | **[-0.915, -0.177]** |  |  | RPCC→LFP~~RAG→LAG | 0.258 | **[0.098, 0.424]** |  |
|  | effTotal | -0.573 | **[-0.829, -0.175]** |  |  | RAG→RFP~~RPCC→LAG | 0.254 | **[0.013, 0.493]** |  |
| Covariance | LPCC→RFP~~RPCC→RFP | 0.882 | **[0.830, 0.928]** |  |  | RFP→LFP~~LPCC→LAG | 0.147 | **[0.040, 0.250]** |  |
|  | LPCC→LFP~~LPCC→RFP | 0.881 | **[0.817, 0.931]** |  |  | LPCC→LFP~~RAG→LAG | 0.244 | **[0.066, 0.422]** |  |
|  | LPCC→LAG~~RPCC→LAG | 0.89 | **[0.839, 0.928]** |  |  | RAG→RFP~~LPCC→LAG | 0.194 | [-0.034, 0.436] |  |
|  | LAG→LFP~~LAG→RFP | 0.875 | **[0.793, 0.932]** |  |  | LFP→RFP~~RPCC→LAG | 0.071 | [-0.044, 0.201] |  |
|  | RPCC→LFP~~RPCC→RFP | 0.87 | **[0.816, 0.916]** |  |  | RFP→LFP~~RAG→LAG | 0.151 | **[0.055, 0.257]** |  |
|  | RAG→LFP~~RAG→RFP | 0.861 | **[0.785, 0.919]** |  |  | PS_AudInc_~~PS_VisCom_ | -0.187 | [-0.913, 0.140] |  |
|  | RFP→LFP~~LFP→RFP | 0.857 | **[0.785, 0.913]** |  |  | PS_AudCom_~~PS_VisInc_ | -0.025 | [-0.633, 0.381] |  |
|  | LPCC→LFP~~RPCC→LFP | 0.844 | **[0.776, 0.903]** |  | Variance | PS_AudCom_~~PS_AudCom_ | 0.574 | [-0.284, 0.955] |  |
|  | RPCC→LFP~~LPCC→RFP | 0.752 | **[0.651, 0.832]** |  |  | PS_AudInc_~~PS_AudInc_ | 0.583 | **[0.011, 0.931]** |  |
|  | LPCC→LFP~~RPCC→RFP | 0.749 | **[0.667, 0.823]** |  |  | PS_VisCom_~~PS_VisCom_ | 0.422 | [-0.897, 0.938] |  |
|  | RAG→RFP~~RPCC→RFP | 0.64 | **[0.478, 0.773]** |  |  | PS_VisInc_~~PS_VisInc_ | 0.324 | [-0.864, 0.823] |  |
|  | RFP→LFP~~RPCC→LFP | 0.624 | **[0.477, 0.752]** |  |  | RFP→LFP~~RFP→LFP | 0.835 | **[0.694, 0.953]** |  |
|  | LAG→RFP~~RPCC→RFP | 0.634 | **[0.479, 0.772]** |  |  | LAG→LFP~~LAG→LFP | 0.934 | **[0.824, 0.998]** |  |
|  | LFP→RFP~~LAG→RFP | 0.647 | **[0.490, 0.770]** |  |  | RAG→LFP~~RAG→LFP | 0.894 | **[0.778, 0.981]** |  |
|  | LAG→RFP~~LPCC→RFP | 0.623 | **[0.484, 0.751]** |  |  | LPCC→LFP~~LPCC→LFP | 0.89 | **[0.769, 0.983]** |  |
|  | LFP→RFP~~RPCC→RFP | 0.61 | **[0.477, 0.732]** |  |  | RPCC→LFP~~RPCC→LFP | 0.857 | **[0.727, 0.969]** |  |
|  | LPCC→LFP~~LPCC→LAG | 0.582 | **[0.383, 0.765]** |  |  | LFP→RFP~~LFP→RFP | 0.847 | **[0.718, 0.957]** |  |
|  | LPCC→LFP~~LAG→RFP | 0.598 | **[0.451, 0.730]** |  |  | LAG→RFP~~LAG→RFP | 0.919 | **[0.803, 0.995]** |  |
|  | RPCC→LFP~~LFP→RFP | 0.584 | **[0.441, 0.702]** |  |  | RAG→RFP~~RAG→RFP | 0.912 | **[0.802, 0.993]** |  |
|  | LAG→LFP~~LPCC→LFP | 0.613 | **[0.432, 0.764]** |  |  | LPCC→RFP~~LPCC→RFP | 0.901 | **[0.771, 0.992]** |  |
|  | RPCC→LFP~~LAG→RFP | 0.596 | **[0.437, 0.738]** |  |  | RPCC→RFP~~RPCC→RFP | 0.896 | **[0.761, 0.992]** |  |
|  | LAG→LFP~~RPCC→LFP | 0.609 | **[0.431, 0.767]** |  |  | RAG→LAG~~RAG→LAG | 0.939 | **[0.835, 0.998]** |  |
|  | RPCC→LFP~~RPCC→LAG | 0.557 | **[0.431, 0.674]** |  |  | LPCC→LAG~~LPCC→LAG | 0.958 | **[0.876, 1.000]** |  |
|  | RFP→LFP~~LAG→LFP | 0.606 | **[0.428, 0.745]** |  |  | RPCC→LAG~~RPCC→LAG | 0.935 | **[0.822, 0.998]** |  |
|  | RAG→LFP~~RPCC→LFP | 0.599 | **[0.426, 0.738]** |  |  | PS~~PS | 0.349 | [-0.088, 0.846] |  |
|  | RFP→LFP~~RPCC→RFP | 0.583 | **[0.441, 0.714]** |  |  | Age~~Age | 1 | **[1.000, 1.000]** |  |

Regional intra-network EC revealed an interesting pattern (Figure 5). While still less than half of those EC pathways demonstrated age-related changes, those significant ones within SN (4/12), within DAN (5/12) and within CN (3/6) were increased with age, while those within DMN (13/30) were reduced among older participants.

## Goodness of Fit

Several goodness-of-fit tests were conducted on all SEM models [6]. The Chi-square test with a p-value greater than 0.05, Standardised Root Mean Square Residual (SRMR) less than 0.08, Comparative fit index (CFI) greater than 0.9 and Root Mean Square Error of Approximation (RMSEA) less than 0.08 indicates acceptable fit [7-9]. All reported models showed good model fit (Supp. Table 2).

*Supplementary Table 8 Goodness of fit tests*

| Within/Between | χ2 | df | p-value | CFI | SRMR | RMSEA |
| --- | --- | --- | --- | --- | --- | --- |
| Inter-network | 17.64 | 12.00 | **0.127** | **0.977** | **0.053** | **0.075** |
| Intra-SN | 10.49 | 13.00 | 0.654 | 1.000 | 0.024 | 0.000 |
| Intra-CN | 3.45 | 10.00 | 0.969 | 1.000 | 0.024 | 0.000 |
| Intra-DAN | 14.67 | 16.00 | 0.549 | 1.000 | 0.025 | 0.000 |
| Intra-DMN | 27.67 | 44.00 | 0.974 | 1.000 | 0.071 | 0.000 |

†: Model was not built as not built as no and only one a-path were significant with 95% confidence interval for DAN→SN and DAN→DMN respectively.

p-value: p-value of the Chi-squared test, bolded values were greater than 0.05. CFI: Comparative fit index, bolded values were greater than 0.9. SRMR: Standardised Root Mean Squared Residual, bolded values were smaller than 0.08. RMSEA: Root Mean Squared Error of Approximation, bolded values were smaller than 0.08. Pos: Positive mediator. Neg: Negative mediator.

*Supplementary Table 9 Age-corrected Mean Intrinsic Connectivity*

| **Path** | **E** | **Mean** | **95% Confidence Interval** | | **p** | **fdr-p** |
| --- | --- | --- | --- | --- | --- | --- |
|  |  |  | **Lower** | **Upper** |  |  |
| DAN→CN |  | 0.9995 | 0.9986 | 1.0003 | 0.2164 | 0.6492 |
| DAN→DN |  | 0.9989 | 0.9977 | 1.0000 | 0.0572 | 0.2288 |
| DAN→SN | Excitatory | 1.0069 | 1.0057 | 1.0082 | <0.0001 | <0.0001 |
| CN→DAN | Excitatory | 1.0086 | 1.0075 | 1.0099 | <0.0001 | <0.0001 |
| CN→DMN | Excitatory | 1.0033 | 1.0023 | 1.0043 | <0.0001 | <0.0001 |
| CN→SN | Excitatory | 1.0064 | 1.0053 | 1.0074 | <0.0001 | <0.0001 |
| DMN→DAN | Inhibitory | 0.9969 | 0.9953 | 0.9984 | 0.0002 | 0.0014 |
| DMN→CN |  | 0.9998 | 0.9986 | 1.0010 | 0.7930 | 0.7930 |
| DMN→SN | Inhibitory | 0.9935 | 0.9912 | 0.9956 | 0.0002 | 0.0014 |
| SN→DAN | Excitatory | 1.0074 | 1.0061 | 1.0087 | <0.0001 | <0.0001 |
| SN→CN | Inhibitory | 0.9991 | 0.9983 | 0.9999 | 0.0296 | 0.1480 |
| SN→DMN |  | 0.9994 | 0.9981 | 1.0007 | 0.3632 | 0.7264 |

Note: The connectivity indices were extracted from the A-matrix of the subject-level rDCM models that represented the intrinsic connectivity. Connectivity values smaller than 1 indicate an inhibitory influence, whereas greater than 1 an excitatory influence. The confidence intervals and p-values were established with 5000 bootstraps. The p-values were calculated as bootstrapped one-sample t-test against 1. FDR corrected p-values smaller than 0.05 were regarded as significant.

References

1. Kelly RE, Alexopoulos GS, Wang Z *et al.* Visual inspection of independent components: defining a procedure for artifact removal from fMRI data. J Neurosci Methods 2010; 189: 233–45.

2. Avants BB, Epstein CL, Grossman M, Gee JC. Symmetric diffeomorphic image registration with cross-correlation: Evaluating automated labeling of elderly and neurodegenerative brain. Med Image Anal 2008; 12: 26–41.

3. Razlighi QR, Habeck C, Barulli D, Stern Y. Cognitive neuroscience neuroimaging repository for the adult lifespan. Neuroimage 2017; 144: 294–298.

4. Jung K, Friston KJ, Pae C *et al.* Effective connectivity during working memory and resting states: A DCM study. Neuroimage 2018; 169: 485–495.

5. Yoon M, Kim ES. A comparison of sequential and nonsequential specification searches in testing factorial invariance. Behav Res Methods 2014; 46: 1199–1206.

6. Schermelleh-Engel K, Moosbrugger H, Müller H. Evaluating the Fit of Structural Equation Models: Tests of Significance and Descriptive Goodness-of-Fit Measures. Methods of Psychological Research 2003; 2: 23–74.

7. Hu L, Bentler PM. Cutoff criteria for fit indexes in covariance structure analysis: Conventional criteria versus new alternatives. Struct Equ Model Multidiscip J 1999; 6: Jan-55.

8. McDonald RP, Ho M-HR. Principles and Practice in Reporting Structural Equation Analyses. Psychol Methods 2002; 7: 64–82.

9. Bentler PM, Bonett DG. Significance tests and goodness of fit in the analysis of covariance structures. Psychol Bull 1980; 88: 588–606.
